# Adverse obstetric and perinatal outcomes among Palestinian adolescent mothers in the West Bank: a retrospective cohort study

**DOI:** 10.3389/fgwh.2025.1732760

**Published:** 2026-01-09

**Authors:** Eman Bellah Khamaysa, Hadil Masri, Sahar Hassan

**Affiliations:** 1 Master’s Program in Women’s Health, Faculty of Pharmacy, Nursing and Health Professions, Birzeit University, Birzeit, Palestine; 2 Women’s Health and Development Unit, Ministry of Health, Ramallah, Palestine; 3 Master’s Program in Women’s Health and Department of Nursing, Faculty of Pharmacy, Nursing and Health Professions, Birzeit University, Birzeit, Palestine

**Keywords:** adolescent pregnancy, adolescents, adverse obstetric outcomes, adverse perinatal outcomes, occupied Palestinians territory

## Abstract

**Background:**

It is estimated that 13% of adolescent girls globally give birth before the age of 18. Several studies have shown that pre-eclampsia, eclampsia, puerperal infections, preterm birth, low birth weight, and neonatal death are more common in adolescent mothers than in adult mothers. This study aimed to investigate whether adolescent pregnancy increases the risk of adverse obstetric and perinatal outcomes compared with pregnancies among women aged 20–35 years in the West Bank, occupied Palestinian territory.

**Methods:**

A retrospective cohort study utilizing data extracted from the electronic health information system database. The study population comprised 11,189 women who gave birth in three governmental hospitals in the West Bank during the year 2022, of whom 762 (6.8%) were aged ≤19 years. Differences between the two age groups were analyzed using chi-square test (*χ*^2^) for categorical variables and independent student test for continuous variables. Multiple logistic regression and sensitivity analyses were performed to adjust for confounders and to examine the association between the adolescent age group and adverse obstetric and perinatal outcomes.

**Results:**

Most adolescent mothers were primiparous (82.2%) and had a singleton pregnancy (98.2%). Adolescent mothers were more likely to experience preterm birth (aOR 1.568, CI 95% 1.262–1.949, *p* < 0.001) and low birth weight (aOR 1.657, CI 1.327–2.068, *p* < 0.001). Low Apgar score at first minute was demonstrated among primiparous adolescents (aOR 1.774, CI 1.149–2.737, *p* = 0.01). While adolescent primiparous mothers were less likely to deliver by cesarean section compared to older mothers (aOR 0.270, CI 0.212–0.342, *p* = 0.00), fetal malpresentation as an indication for cesarean section was higher among adolescents (aOR 2.150, CI 1.329–3.479, *p* = 0.002). No significant differences were observed between the two age groups in terms of gestational diabetes, hypertensive disorders, antepartum or postpartum hemorrhage, blood transfusion, induction of labor, instrumental delivery, five-minute Apgar score, admission to neonatal intensive care unit, neonatal malformation, or stillbirth.

**Conclusion:**

Adolescent pregnancies should be classified as high-risk given their association with multiple adverse obstetric and perinatal outcomes. These findings underscore the need for policymakers to strengthen preventive strategies and to ensure strict enforcement of the child marriage prohibition law.

## 1 Introduction

Adolescent pregnancy, as defined by the World Health Organization (WHO), is a woman who gets pregnant at any age between 10 and 19 years (1). Globally, it is estimated that 13% of adolescent girls give birth before the age of 18 (2). This phenomena is quite common in low-middle-income countries (LMICs) (3). In the LMICs, where adolescent pregnancy reaches 21 million girls, 12 million of them give birth between the ages of 15 and 19 years (1). Pregnancy makes adolescent girls more vulnerable to unfavorable health consequences due to their physical immaturity (2). Indeed, maternal conditions stand as one of the top causes of death and disabilities among adolescents aged 15–19 years worldwide (2). Moreover, global maternal mortality is estimated at 260 per 100,000 live births among adolescents aged 15–19, compared to 190 per 100,000 live births among older women aged 20–24 (4). Africa and Eastern Mediterranean regions have the highest maternal mortality ratios worldwide, at 570 and 430 per 100,000 live births, respectively. In Sub-Saharan Africa, it was reported that 20% of all maternal deaths were among adolescents, where the main death causes were unsafe abortion, pre-eclampsia, and hypertensive disorders (4).

In general, pregnancy at this age puts women at higher risk of abortion, preterm birth, low birth weight (LBW), hypertensive disorders, and puerperal endometritis in addition to increased neonatal illness and death (5–11). Nevertheless, these outcomes can vary according to the context and country (12). A large multi-country population-based study including 269,273 women in six LMICs, found that adolescent pregnancies among girls aged 15–19 were associated with significantly higher risks of preterm birth and LBW (13). A secondary analysis of cross-sectional data from 29-country study across Africa, Latin America, Asia and the Middle East, reported higher odds for hypertensive disorders, infections, LBW, and preterm birth among adolescent mothers (7). On the other hand, in high-income countries, the high quality and comprehensive coverage of maternal care including advanced technology, early booking, and adequate antenatal follow-up result in adolescents having maternal and neonatal outcomes comparable to those of older mothers (12, 14, 15).

Despite that there is a growing body of evidence from LMICs indicating that adolescent pregnancy is associated with adverse maternal and perinatal outcomes, adolescents in the LMICs and the eastern Mediterranean regions still have high prevalence of adolescence pregnancy, unmet need for contraception, unintended pregnancies, and increased risk for maternal mortality and morbidity (10, 11, 16, 17). Humanitarian Crises and ongoing conflict, such as in the occupied Palestinians territory (oPt), further exacerbate the adolescent mothers' vulnerability due to limited access to adequate health infrastructure, increased maternal and neonatal complications, and the lack of policies and guidelines addressing adolescent sexual and reproductive health (18–24). Recent studies exploring adolescents' health in the oPt during the current escalation of violence, consistently show that adolescents face profound and interconnected health challenges (18–24). These challenges include significant gaps in access to sexual and reproductive health information and youth-appropriate services, rising risks of early marriage and adolescent pregnancy (19, 23, 24). In addition, the war in Gaza has placed adolescents under chronic stress, repeated trauma exposure, and extremely limited availability of psychological support (20, 24). The conflict has further disrupted their daily lives through large-scale displacement, the loss of family and community support networks, and major interruptions to education, nutrition, and basic health care (18–20, 23, 24). Within this fragile and conflict-affected context, damage to health infrastructure, shortages of essential supplies, and severe movement restrictions undermine adolescents' ability to obtain timely, and continuous care.

Despite the child marriage prohibition act, approximately, 22% of Palestinian women were married before the age of 18 in 2019–2020 (25). During the same period, 4.3% of adolescents aged 15–19 had live births, and 5.2% had a live birth or were pregnant for the first time (25). While evidence highlights the vulnerabilities of adolescents in the oPt, there is a critical lack of research examining the specific maternal and neonatal outcomes associated with adolescent pregnancies. Most existing studies focus on broader health risks or service access, with limited data quantifying complications such as preterm birth, LBW, or postpartum hemorrhage in this population. This knowledge gap is particularly pronounced in conflict-affected and humanitarian settings, where systemic barriers and instability may further exacerbate health risks. Given the challenging situation and restricted resources, it is imperative to highlight the burden of this problem to inform clinical guidance, targeted interventions, and health system strengthening for adolescent mothers in fragile contexts. The aim of this study is to investigate the obstetric and perinatal outcomes among adolescent mothers compared with the older maternal age group (20–35 years) in the Palestinian context.

## 2 Materials and methods

### 2.1 Study design and population

This retrospective cohort study analyzed a total of 11,189 women aged ≤35 years. Three main governmental hospitals in the West Bank were selected based on the large number of annual births and their geographical location (North, Middle, and South) across the West Bank. In addition, these hospitals are among the major obstetrical care providers with the highest number of obstetric admissions compared to other governmental hospitals in the West Bank.

The study population comprised women who gave birth in the three selected hospitals during the year 2022, from January 1 to December 31, and all women who were less than 35 years old, gave birth at gestational age ≥24 weeks or delivered a baby of birthweight ≥500 g, and had complete data were included. Women older than 35 years were excluded because pregnancy at this age is more likely to be associated with complications. The study population was categorized according to maternal age into two groups: ≤19 years (adolescent mothers) and 20–35 years (older mothers), with the latter used as a reference group.

### 2.2 Data source and variables

The information technology (IT) department at the MoH extracted the data for all women who gave birth during 2022 at the selected hospitals from the health information system database. The dataset included variables on women's current pregnancy and obstetric conditions, medical problems, delivery circumstances, and newborn outcomes.

The data received as ready-to-use variables and text data notes on excel sheet that was converted to SPSS file. The data was reviewed numerous times to find any potential data quality problems like outliers, missing values, duplicate entries, and incorrect data format. The missing values were identified as blank cells. The duplicate cases were detected by the women's ID. Outliers were also addressed by identifying the data points that deviated from the rest of the data. These were corrected or confirmed based on the verification with other variables. The data included 13,054 cases; 1,700 cases were removed for not meeting the inclusion criteria due to maternal age greater than 35 years. An additional 175 cases were removed because of missing value or duplication. The final sample consisted of 11,189 cases that met all inclusion criteria.

The dataset was standardized and re-checked for consistency across the variables; the categorical variables were standardized as (1) for the presence of the outcome and (0) for the absence of the outcome.

The main obstetric outcomes that were studied included preterm birth (birth at less than 37 weeks), gestational hypertension, preeclampsia, eclampsia, gestational diabetes, blood transfusion, mode of delivery (spontaneous vaginal delivery (SVD), vacuum delivery, and cesarean section (CS), indications of CS, antepartum hemorrhage, postpartum hemorrhage, and induction of labor. The main perinatal outcomes included LBW (Birthweight less than 2,500 g), low Apgar score (less than 7) at first and five minutes, NICU admission, stillbirth, and neonatal malformation. The data was reviewed numerous times and checked for consistency across the variables. The text notes were subsequently utilized to verify the accuracy of the data and to complete some variables.

A preliminary analysis was conducted to ensure the validity of the data. This included descriptive summaries such as minimum and maximum values, percentages, and frequencies. Histograms were used to check the distribution of continuous variables before standard categorization, and bivariate analysis was conducted to further explore the data. This process was repeated as needed until the required quality of the dataset for analysis was achieved. The final cleaned dataset was reviewed by second researcher.

### 2.3 Statistical analysis

The data were analyzed using SPSS version 26. Maternal characteristics were described by using mean and standard deviation (SD) for continuous variables, and frequencies and percentages (%) were used to summarize categorical variables. Differences between the adolescent and older reference age groups were assessed by using chi-square test (*χ*2) or Fisher's Exact test (if cell count is less than 5) for categorical variables, and independent student test used for continuous variables. Multiple logistic regression was performed to examine the association between adolescent and adverse obstetric and perinatal outcomes after adjustment for maternal characteristics and medical and obstetric problems as confounding variables. The findings were presented as unadjusted and adjusted odds ratio (aOR). The significance level was set at 0.05, with a CI of 95%. To assess the robustness of our findings, the data was stratified by parity (primiparous and multiparous) and all logistic analysis was repeated per stratum. Furthermore, sensitivity analysis was conducted to demonstrate the independent role of maternal age; interaction terms for age by parity and age by comorbidities were added to the logistic regression model to explore effect modification.

### 2.4 Ethical statement

The study was approved by the research ethical committee at the faculty of pharmacy, nursing, and health professions at Birzeit University (BZUPNH 2303). We obtained permission from the Palestinian Ministry of Health to facilitate data retrieval and utilization.

Our study is non- interventional and does not carry any potential harms or risks but is designed to drive quality improvement. No consent obtained since we did not prospectively enroll the women and only managed electronic available data retrospectively. The privacy and confidentiality of women's information maintained by using login credentials and password-protected documents. Final data file deanonymized by deleting file ID numbers.

## 3 Results

A total of 11,189 women were included in the study, 762 (6.8%) of whom were ≤19 (adolescent mothers), with an age range between 14 and 19 years old (mean age of 18.1, SD ± 1.07). Most of the adolescent women were primiparous compared to the older age group (82.2% vs. 35.1%, *p* < 0.001). The mean gestational age at delivery for the adolescent group was lower than the older group (38.4 vs. 38.7, *p* < 0.001).

Maternal characteristics in the study are presented in (Table 1).

**Table 1:** Descriptive analysis of maternal characteristics.

Maternal characteristics	Total (14–35) years 11,189 (100%)	Adolescent group (≤19) years 762 (6.8%)	Older age group (20–35) years 10,427 (93.2%)	*P* value (CI 95%)
Maternal age (years) (mean, SD)	25.7 ± 4.5	18.1 ± 1.07	26.3 ± 4.2	<0.001 (7.875–8.467)
Gestational age at delivery (weeks) (mean, SD)	38.68 ± 2.0	38.38 ± 2.5	38.71 ± 2.0	<0.001 (0.172–0.479)
Type of gestation, *n* (%)				
Singleton	10,924 (97.6)	748 (98.2%)	10,176 (97.6%)	0.310 (0.765–2.269)
Multiple	265 (2.4%)	14 (1.8%)	251 (2.4%)	
Parity, *n* (%)				
Primipara	4,282 (38.3%)	626 (82.2%)	3,656 (35.1%)	<0.001 (7.051–10.306)
Multipara	6,907 (61.7%)	136 (17.8%)	6,771 (64.9%)	
Medical problems, *n* (%)	82 (0.7)	1 (0.1%)	81 (0.7)	0.340* (0.797–41.28)

*N*: number of cases, (%): percentage of cases, SD: standard deviation.

* Fisher's Exact test, Medical problems include chronic hypertension or diabetes mellitus (the presence of one of them considered yes).

Table 2 presents the obstetric conditions and outcomes in each age group. Adolescent mothers had a higher prevalence of preterm birth compared to older mothers (15.9% vs. 10%, *p* < 0.001). No significant differences were observed between adolescent and older mothers in the prevalence of gestational diabetes, hypertensive disorders, antepartum hemorrhage, postpartum hemorrhage, induction of labor, and blood transfusions (Table 2).

**Table 2:** Obstetric outcomes per maternal age group.

	Age group		
Obstetric outcomes	(≤19 years) 762 (6.8%) *n* (%)	(20–35) 10,427 (93.2%) *n* (%)	*P* value (CI 95%)
Preterm birth	121 (15.9%)	1,045 (10%)	0.001 (1.381–2.079)
Gestational diabetes	2 (0.3%)	37 (0.3%)	1.000* (0.178–3.072)
Gestational hypertension	3 (0.4%)	96 (0.9%)	0.134 (0.134–1.345)
Preeclampsia	13 (1.7%)	185 (1.8%)	0.921 (0.545–1.694)
Eclampsia	3 (0.4%)	32 (0.3%)	0.731*(0.392–1.203)
Antepartum haemorrhage	9 (1.2%)	136 (1.3%)	0.772 (0.455–1.769)
Postpartum haemorrhage	11 (1.4%)	103 (1%)	0.227 (0.785–2.746)
Blood transfusion	4 (0.5%)	61 (0.6%)	1.000*(0.325–2.473)
Induction of labour	71 (9.3%)	930 (8.9%)	0.710 (0.814–1.352)
Mode of delivery			
Caesarean section	99 (13%)	2,089 (20%)	<0.001 (0.480–0.740)
Vacuum delivery	27 (3.5%)	188 (1.8%)	0.002 (1.327–3.015)
Spontaneous vaginal delivery	636 (83.5%)	8,150 (78.2%)	<0.001 (1.158–1.717)

*N*: number of cases, %: percentage of cases.

* Fisher exact test.

Regarding the mode of delivery, adolescent mothers were more likely to deliver by SVD compared to older mothers (83.5% vs. 78.2%, *p* < 0.001), whereas CS rates were significantly higher among the older group (20% vs. 13%, *p* < 0.001). Sub-analysis of CS indications revealed that fetal malpresentation was significantly more frequent among the adolescent mothers compared to older mothers (30% vs. 19.7%, *p* = 0.018). Conversely, older mothers were more likely to undergo CS due to previous CS than adolescents (42.1% vs. 21.1%, *p* < 0.001). In addition, vacuum-assisted delivery was required more often among adolescent mothers than among older mothers (3.5% vs. 1.8%, *p* = 0.004) (Table 2).

Regarding perinatal outcomes, adolescent mothers had significantly higher prevalence of LBW, low Apgar score at 1 and 5 min, and a greater need for NICU admission compared to older mothers. No significant differences were observed between the two groups in terms of neonatal malformations and stillbirth (Table 3).

**Table 3:** Perinatal outcomes based on age group.

Perinatal outcomes	Age group		*P* value (CI 95%)
	≤19 years 762 (6.8%) *n* (%)	(20–35) 10,427 (93.2%) *n* (%)	
Low birth weight (less than 2,500 g)	118 (15.5%)	927 (8.9%)	<0.001 (1.526–2.311)
Apgar score at 1 min (less than 7)	40 (5.2%)	272 (2.6%)	<0.001 (1.472–2.907)
Apgar score at 5 min (less than 7)	16 (2.1%)	128 (1.2%)	0.039 (1.021–2.917)
Need for NICU	67 (8.8%)	559 (5.4%)	<0.001 (1.306–2.218)
New-born malformation	5 (0.7%)	61 (0.6%)	0.804* (0.450–2.802)
Stillbirth	8 (1%)	72 (0.7%)	0.256 (1.306–2.218)

*N*: number of cases, %: percentage of cases.

* Fisher exact test.

**Figure 1:**
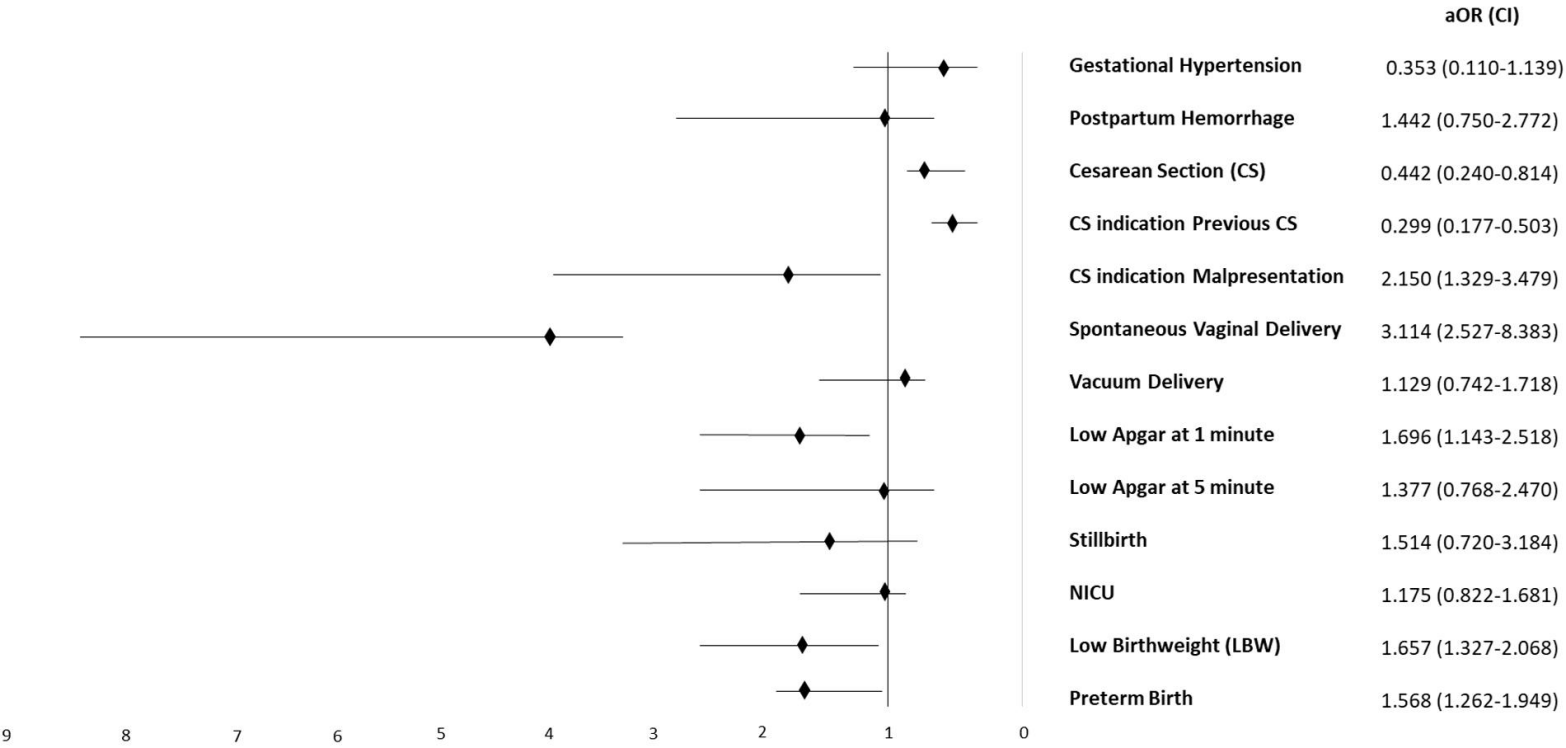
Adjusted odds ratio (aOR) for maternal and neonatal outcomes in adolescent vs. adult mothers.

The multiple logistic regression model (Table 4) showed that the adolescent maternal age was significantly associated with preterm birth (aOR 1.568, CI 95% 1.262–1.949, *p* < 0.001), LBW (aOR 1.657, CI 1.327–2.068, *p* < 0.001), and low Apgar at 1 min (aOR 1.696, CI 1.143–2.518, *p* = 0.009). Even after controlling confounders, the adolescent mothers were less likely to deliver by CS compared to older mothers (aOR 0.442, CI 0.240–0.814, *p* = 0.009), and fetal malpresentation as an indication for CS remained significantly higher among the adolescent mothers (aOR 2.150, CI 1.329–3.479, *p* = 0.002), while vacuum delivery was no longer significant to be associated with the adolescent group when adjusted for parity (aOR 1.129, CI 0.742–1.718, *p* = 0.57). In addition, the low Apgar score at 5 min was non-significant after adding preterm birth to the model (aOR 1.377, CI 0.768–277, CI 0.768–2.470, *p* = 0.283) and babies who needed NICU after delivery was non-significant when low Apgar score was added to the model (aOR 1.175, CI 0.822–1.681, *p* = 0.376). Figure 1 graphically summarizes the aOR and 95% CI of the main outcomes.

**Table 4:** Multiple regression model of obstetric and perinatal outcomes.

	Age group ≤ 19 years			
Maternal and perinatal outcomes	Unadjusted OR (CI 95%)	*P* value (0.05)	Adjusted OR (CI 95%)	*P* value
Preterm birth less than 37 weeks	1.695 (1.381–2.079)	<0.001	1.568 (1.262–1.949)	<0.001
Low birth weight (less than 2,500 g)	1.878 (1.526–2.311)	<0.001	1.657 (1.327–2.068)	<0.001
Apgar score at 1 (less than 7)	2.068 (1.472–2.907)	<0.001	1.696 (1.143–2.518)	0.009
Apgar score at 5 (less than 7)	1.726 (1.021–2.917)	0.042	1.377 (0.768–2.470)	0.283
Need for neonatal intensive care unit	1.702 (1.306–2.218)	<0.001	1.175 (0.822–1.681)	0.376
Mode of delivery				
Caesarean section	0.596 (0.480–0.740)	<0.001	0.442 (0.240–0.814)	0.009
Vacuum delivery	2.001 (1.327–3.015)	0.001	1.129 (0.742–1.718)	0.570
Spontaneous vaginal delivery	1.410 (1.158–1.717)	0.001	3.114 (2.527–8.383)	0.000
Indications for CS				
Mal-presentation	1.744 (1.093–2.783)	0.020	2.150 (1.329–3.479)	0.002
Previous CS	0.368 (0.220–0.617)	0.000	0.299 (0.177–0.503)	0.000
Postpartum haemorrhage	1.468 (0.785–2.746)	0.229	1.442 (0.750–2.772)	0.273
Gestational hypertension	0.425 (0.134–1.345)	0.146	0.353 (0.110–1.139)	0.082
Stillbirth	1.526 (0.732–3.180)	0.259	1.514 (0.720–3.184)	0.274

aOR, adjusted odds ratio; CI, confidence interval.

The stratified analysis indicated that adolescent maternal age was associated with increased odds for preterm birth and LBW among both primiparous and multiparous women, while the association with low Apgar scores and low CS delivery was significant only among primiparous women (Tables 5, 6).

**Table 5:** Regression model of obstetric and perinatal outcomes (primipara).

	Age group ≤19 years			
Maternal and perinatal outcomes	Unadjusted OR (CI 95%)	*P* value (0.05)	Adjusted OR (CI 95%)	*P* value (0.05)
Preterm birth	1.350 (1.064–1.711)	0.013	1.47 (1.132–1.849)	0.003
Low birth weight (less than 2,500 g)	1.390 (1.095–1.765)	0.007	1.384 (1.046–1.831)	0.023
Apgar score at 1 (less than 7)	1.692 (1.145–2.501)	0.008	1.774 (1.149–2.737)	0.01
Apgar score at 5 (less than 7)	2.017 (1.093–3.722)	0.025	0.539 (0.203–1.432)	0.215
Need for neonatal intensive care unit	1.308 (0.969–1.766)	0.079	1.162 (0.787–1.718)	0.450
Mode of delivery				
Caesarean section	0.275 (0.218–0.347)	<0.001	0.270 (0.212–0.342)	0.00
Vacuum delivery	1.093 (0.707–1.690)	0.688	1.941 (0.467–8.075)	0.362
Spontaneous vaginal delivery	3.168 (2.582–3.916)	0.001	3.134 (2.526–3.887)	0.001

aOR, adjusted odds ratio; CI, confidence interval.

**Table 6:** Regression model of obstetric and perinatal outcomes (multipara).

	Age group ≤19 years			
Maternal and perinatal outcomes	Unadjusted OR (CI 95%)	*P* value (0.05)	Adjusted OR (CI 95%)	*P* value
Preterm birth less than 37 weeks	2.052 (1.319–3.285)	0.002	2.302 (1.451–3.654)	0.00
Low birth weight (less than 2,500 g)	2.285 (1.423–3.671)	<0.001	1.948 (1.099–3.453)	0.022
Apgar score at 1 (less than 7)	2.010 (0.873–4.628)	0.101	1.420 (0.568–3.551)	0.453
Apgar score at 5 (less than 7)	1.147 (0.279–4.707)	0.849	0.256 (0.043–1.519)	0.134
Need for neonatal intensive care unit	1.718 (0.893–3.305)	0.105	0.996 (0.425–2.336)	0.993
Mode of delivery				
Caesarean section	0.549 (0.268–1.126)	0.102	0.545 (0.263–1.132)	0.103
Vacuum delivery	1.857 (0.448 -7.694)	0.394	1.111 (0.717–1.721)	0.639
Spontaneous vaginal delivery	1.560 (0.815–2.984)	0.179	1.540 (0.797–2.973)	0.199

aOR, adjusted odds ratio; CI, confidence interval.

In the sensitivity analysis (Tables 7, 8), after adding the age–comorbidity interaction term adolescent maternal age remained an independent predictor of preterm birth (aOR 1.584, *p* < 0.001), LBW (aOR 1. 460, *p* = 0.004), low Apgar score at one minute (aOR 1.715, *p* = 0.007), and lower risk of CS delivery (aOR 0.437, *p* = 0.007). When the age–parity interaction was included, the associations remained significant for preterm birth (aOR 2.321, *p* < 0.001) and LBW (aOR 2.018, *p* = 0.013), but not for Apgar score or CS delivery.

**Table 7:** Multiple logistic regression with sensitivity analysis (age-comorbidity).

Maternal and perinatal outcomes	Adjusted OR (CI 95%)	*P* value
Preterm birth less than 37 weeks	1.584 (1.247–1.970)	0.000
Low birth weight (less than 2,500 g)	1.460 (1.131–1.886)	0.004
Apgar score at 1 (less than 7)	1.715 (1.156–2.544)	0.007
Caesarean section	.437 (.239–.799)	0.007

**Table 8:** Multiple logistic regression with sensitivity analysis (age–parity).

Maternal and perinatal outcomes	Adjusted OR (CI 95%)	*P* value
Preterm birth less than 37 weeks	2.321 (1.463–3.683)	0.000
Low birth weight (less than 2,500 g)	2.018 (1.161–3.508)	0.013
Apgar score at 1 (less than 7)	1.531 (.621–3.775)	0.355
Caesarean section	0.582 (0.282–1.201)	0.143

## 4 Discussion

This study demonstrated that adolescent pregnancy is significantly associated with several adverse obstetric and perinatal outcomes. Adolescent mothers were at higher risk of preterm birth and of delivering LBW infants. In addition, primiparous adolescents had higher odds of low Apgar score at 1 min and were less likely to undergo CS. Given that the oPt is under Israeli occupation, with hundreds of additional checkpoints recently installed across the West Bank, access to healthcare services is a challenge and the delivery of maternal health care is fragmented, unregulated, and varies considerably in quality (26). Access to family planning services is challenging due to limited infrastructure and shortage of staff and commodities (27, 28). In addition, care during childbirth often falls short of professional standards (27, 28). Neonatal care is also suboptimal, as the NICUs, which receive very sick or extremely LBW babies, are often unprepared for their needs due to shortage of skilled staff, equipment, and medications (29). This highlights the additional burden of adolescent pregnancy, which necessitates a close monitoring and follow-up of the adolescents and their babies during the antenatal, intrapartum, and postnatal periods.

In our study, the prevalence of adolescent pregnancy was 6.8%, which was close to the 5.8% reported in a previous study conducted in ten WHO Eastern Mediterranean countries in 2019–2020 (18). The prevalence in oPt was lower than in Egypt, Iraq, Sudan, and Yemen, which were 10.9%, 13.2%, 15.2%, and 10.7%, respectively (18). In comparison, the prevalence in oPt was higher than that in Jordan, Morocco, Tunisia, and Qatar, where it ranged between 0.4% and 5.2% (30). Despite the drop of marriage rate among Palestinian girls younger than 18 years from 24% in 2010 to 19.3% in 2019 (25), child marriage and adolescent pregnancy are still common due to weak enforcement of the Palestinian prohibition of child marriage act (31). This possibly explains, along with other financial, cultural and political factors, the higher prevalence of adolescent pregnancy in the oPt compared to other countries in the region (32). Therefore, there is a need for increased community awareness regarding the adverse consequences of child marriage to increase their commitment to the law based on their motivation rather than fear of breaking the law.

In this study, we found a 60% increase in the odds of preterm birth among adolescent mothers compared to mothers aged 20 to 35, regardless of medical or obstetric conditions. Despite that mean gestational age for both age groups seem clinically comparable (38.38 vs. 38.71, *p* < 0.001), the difference was statistically significant. This relatively similar mean masks the distribution among adolescents, which was skewed toward the extremes, with higher proportions of both preterm and post-term deliveries. The elevated rate of preterm birth among adolescents remains clinically relevant, as it indicates an increased risk of adverse neonatal outcomes. A systematic review and meta-analysis including 59,670,142 adolescent mothers reported that the most common obstetric outcome was preterm birth (33). Many other studies aligned with our finding (34–39). Amoadu M. et al, in their scoping review, analyzed 53 studies from several low- and middle-income countries in Africa, including a total of 263,580 pregnant women, of whom 17.5% were adolescents under 20 years of age (40). They reported an increased risk of preterm birth among adolescent mothers, with those younger than 15 having the highest risk (40). Evidence has also emerged from the Arab region. For example, a retrospective case control study in Oman by Al-Haddabi R. et al, compared singleton primiparous pregnancies among adolescents aged 14–19 years (*n* = 307) with those aged 20–25 years (*n* = 307). The study found that preterm birth before 32 weeks of gestation, was significantly higher in the adolescent group (41). Similarly, studies from Egypt reported elevated rates of preterm birth among adolescent mothers, ranging from 13% to 18% (42, 43). The mechanism of preterm labor among adolescent mothers is still unclear. But, one of the speculations is that the anatomical immaturity like small uterine capacity and short cervical length (44), increase the adolescent mothers' susceptibility to infection-mediated and spontaneous preterm delivery (44). Other factors that can contribute to the risk of preterm birth among adolescent mothers, include inadequate birth spacing, poor antenatal care, and limited education (45). There is good evidence confirming the adverse outcomes of preterm birth and its financial burden on health systems globally, particularly in LMICs (46, 47). Preterm birth is significantly associated with LBW, a low Apgar score at 1 and 5 min, the need for a NICU admission, increased length of hospital stays, and early neonatal death (48, 49). Moreover, preterm birth is a leading cause of death among children under 5 years old (1). Given the restricted resources in the oPt, our study emphasizes the necessity for preventive strategies aiming at reducing the burden of preterm birth.

Literature showed that adolescent mothers were less likely to undergo CS (34, 39, 41, 50–54). Similarly in our study, the initial adjusted logistic regression model demonstrated that adolescent mothers had significantly lower odds for CS compared with older mothers. However, after adding the age-parity interaction, this association was no longer significant, indicating that parity modifies the effect of maternal age. Stratified analysis supported this interaction showing that among primiparous women, adolescent mothers had significantly lower odds of CS compared with older mothers, whereas no significant difference was observed among multiparous women. This suggests that adolescent maternal age reduces the CS risk only among first-time mothers. It might be argued that it is a well-established obstetric pattern that parity increases with maternal age, and the high proportion of primiparous mothers among adolescents (82%) in our study, partly explains their lower CS rates. However, this factor alone does not account for the observed difference. Even after considering parity, adolescent mothers still had lower cesarean rates than older mothers within the same primiparous group. This finding aligns with evidence from low- and middle-income countries, where nulliparous adolescent mothers similarly demonstrate lower likelihood of CS delivery (55). In oPt, the CS delivery rate has been steadily increasing reaching a total 32% (56). However, a considerable variation in CS rates is observed across different hospitals ranging between 17% and 57% (56). Even among governmental hospitals, the rate varies between 17.8% and 42.7% (56). This variation may be attributed to inconsistent adherence to obstetric guidelines and different policies within each facility (57). In light of this, factors other than clinical characteristics and parity may explain the variation in CS rates between adolescent and older mothers.

To understand the underlying causes of CS among our study population, we examined the main indications for CS. We observed that the most common indication associated with adolescence was fetal malpresentation. This finding could be explained by the higher rates of preterm birth and LBW in addition to that adolescents are biologically and physically immature, with incomplete pelvic development, which increases the risk of abnormal fetal lie and malpresentation (58). Although only a limited number of studies have examined the underlying reasons for CS delivery specifically in this age group, most of the available studies report similar patterns, reinforcing the plausibility of this association (51, 55, 58–62). However, a recent cross-sectional study in Vietnam showed that adolescents had have lower percentage of malpresentation babies, while having non-reassuring fetal condition and arrested labor as the main CS indications (63). Several factors may help explain this discrepancy. First, the diagnosis of both conditions, particularly non-reassuring fetal condition, is highly subjective and often depends on clinicians' interpretation of non-reassuring fetal heart rate patterns, which can vary widely between individuals. Second, differences in clinical practice thresholds, such as when to proceed to CS and the criteria used to define arrested labor may not be fully standardized across settings. These variations in clinical definitions, assessment, and practice likely contribute to the differing indications for CS reported by other studies and highlight the need for further research using standardized obstetric criteria.

On the other hand, we found that previous CS, as a main indication for CS, was higher among older mothers than adolescents, similar to the findings reported by previous studies (51, 54, 61).

Adolescent mothers showed two-times increase in the risk of vacuum delivery more than older mothers. However, when parity was added to the regression model, the association was no longer significant, as primiparity confounded this association. In a study by Elci et al. including 2,041 adolescents and 28,233 older mothers in control group, the association of instrumental delivery with maternal age was diminished after adjustment of confounders, including parity (50). Yet, this remains clinically significant, as the majority of adolescent mothers are primiparous and hence at higher risk for instrumental delivery (64, 65). On the other hand, other studies found no difference in instrumental delivery between the two age groups (15, 50, 66, 67). This contradiction may be attributed to differences in the studies' clinical settings and indications for instrumental delivery.

In our study, we did not find an association between maternal age and all categories of hypertensive disorders of pregnancy, gestational diabetes, postpartum hemorrhage, or induction of labor. The lower incidence of these conditions observed in our study, particularly gestational diabetes, may partly reflect the characteristics of our dataset, which included only hospital-based data and was not linked to antenatal health records. Many of these conditions are initially detected and managed at the primary healthcare level, which could lead to underestimation in hospital-based datasets. Furthermore, a few outcome variables may be affected by limitations in documentation practices. For example, some postpartum hemorrhage cases are diagnosed retrospectively, and clinical reporting may be incomplete. Nevertheless, our findings add to the debate in the literature about the association of maternal age with hypertensive disorders in pregnancy. A systematic review and meta-analysis of thirty eight studies involving 20,768 adolescent mothers and 59,481 older mothers in the control group, showed that maternal age has no correlation with all hypertensive disorders of pregnancy (9). In contrast, evidence from large multi-country secondary analyses conducted across ten regions in sub-Saharan Africa, India, and Haiti indicated that adolescents in LMIC settings experience a markedly higher risk of hypertensive disorders (68). Adolescent pregnancies accounted for 34% of all eclampsia cases, with a relative risk ranging from 1.5 to 3.4 compared with older mothers (68). Similarly, a review of studies from several countries, including Egypt, reported consistently higher risks of pre-eclampsia and eclampsia among adolescents (40). However, these findings should be interpreted with caution, as several of the included studies did not adjust for key confounders (66, 67, 69, 70).

In line with available evidence worldwide including, multicounty studies in LMICs and the Arab region (40, 55, 66, 67, 71–74), our study showed a significantly increased risk of LBW among the adolescent mothers. Even after adjusting for preterm birth, as a primary cause of LBW, the adolescent pregnancy remained an independent predicting factor for LBW. This may be explained by the physical immaturity of adolescent mothers in addition to environmental factors such as smoking and malnutrition (75). Babies with LBW are prone to greater health risks like hypothermia, hypoglycemia, malnutrition, neonatal death, and recurrent infections, which require close management and prolonged hospitalization (76). Therefore, this study highlights the need for interventions to mitigate the risk of LBW among adolescent mothers and its associated consequences.

Apgar score is a method to report the status of the newborn after delivery and can be a predictor of the necessity of immediate intervention such as neonatal resuscitation and NICU admission (77). We found that the adolescent group has 70% more odds of a low Apgar score at 1 min, while the risk of a low Apgar score at five minutes diminished after adjusting for preterm birth. After stratifying the analysis by parity, the association between maternal age and low Apgar score at 1 min remained significant only among primiparous. This suggests that adolescents at their first delivery face greater physiological and obstetric challenges such as smaller pelvic dimensions, reduced ability to cope with labor stress, and increased rate of instrumental delivery, which may lead to unfavorable neonatal adaptation. However, the fact that most of the adolescents in our study are primiparous may have influenced the statistical power to detect differences between the two age groups among the parous women. Indeed, the first minute after birth is called the “golden minute” which requires optimal practices, focused observation, and timely management (78). Since midwives and physicians are the primary providers of immediate care for newborns, they must be well-trained and highly qualified, especially in substandard and resource-restricted settings like oPt (79). Some studies have also reported lower Apgar scores among babies born to adolescent mothers (63, 70). For example, a descriptive study from Egypt by Said Faraj and Hassan (70), found lower scores at both 1 and 5 min, while a study from Vietnam by Lam T. et al. (63), reported lower scores specifically at 1 min. Other studies, found no difference between the two age groups (66, 67, 80). Variations related to perinatal care may be a plausible explanation for the contradicting findings related to the Apgar score.

Melekoglu & Sarac (71) found a significant association between adolescent pregnancy and neonatal admission to NICU, and reported that the main reason for admission was hypoglycemia. In our study, we observed a higher prevalence of NICU admission among babies born to adolescent mothers, with a significant association evident in the unadjusted logistic regression model. However, this association disappeared after we adjusted for the Apgar score. A low Apgar score of <7 is one of the predictors for NICU admission (81). Babies born to adolescent mothers in our study had lower Apgar score at 1 min, which likely explains their higher need for NICU admission (50). The high prevalence of NICU admissions among babies of adolescent mothers places an additional psychosocial and financial burden on families, the government, and health systems (47, 82, 83).

## 5 Strengths and limitations

To our knowledge, this study is the first to address the obstetric and perinatal outcomes among adolescent mothers in the West Bank, oPt. Therefore, it provides a basic understanding of the problem and may be used as a benchmark to highlight the risks and burdens of adolescent pregnancy. We included a large number of women in a short period without incurring any cost. The study population is representative as it includes women with various characteristics from three major hospitals across different geographical regions. On the other hand, there are a few limitations of our study that should be taken into consideration. As a retrospective study, it does not establish causality but rather observes possible associations between different variables. Yet, retrospective designs are time- and resource-efficient and feasible in resource- restricted settings, particularly when the exposure and outcome are rare. Additionally, we missed some data about the indications for CS, which may have contributed to our inability to fully explain the differences in CS rates between the two age groups. However, our aim was to get a general idea of whether the indications for CS differ among adolescent mothers. Our study is limited to hospital-based records and is not linked to antenatal or primary care data. As a result, the prevalence of some medical conditions that might be considered as potential confounders such as anemia, diabetes, and thyroid disease maybe slightly underestimated in our dataset. The dataset also, lacked data on smoking, maternal weight, nutritional status, and the number of antenatal visits. Yet, we believe that this has no or little impact on the studied core outcomes, as the most essential variables and recognized confounders were available. Although some outcomes, such as diabetes, hypertensive disorders, and postpartum hemorrhage may be underreported or retrospectively documented, we believe this is unlikely to substantially affect the study's main findings. The key exposures and outcomes are reliably captured in hospital records, and the most essential confounders were included in the analyses. Furthermore, any underreporting is likely non-differential, which would bias associations toward the null rather than overestimate them. Nevertheless, these limitations highlight the importance of future studies integrating multiple data sources to more accurately assess complex maternal and neonatal outcomes.

## 6 Conclusion

Adolescent pregnancies should be classified as high-risk due to their associated adverse obstetric and perinatal outcomes. Our findings indicated a significant association between adolescent pregnancy and preterm birth, LBW, low Apgar score at 1 min, and malpresentation as an indication for CS. These results carry important implications not only for adolescent mothers and their families but also for the healthcare system, particularly in a low-resource, protracted humanitarian and conflict-affected context such as the oPt. The evidence presented should inform policymakers, stakeholders, and healthcare providers highlighting the need for actionable and context-appropriate preventive interventions. Priority actions include enforcing a strict implementation of the child marriage prohibition law, promoting pre-marriage counseling services, and improving adolescents' access to contraception. Additionally, raising awareness among healthcare providers, adolescent girls, families, and communities about the negative consequences of adolescent marriage is crucial. This awareness should be integrated into community-based health education, comprehensive sexuality education in schools, and health-promotion programs delivered through primary healthcare centers, all of which can serve as a preventive measure to mitigate the adverse effects on individuals, society, and the healthcare system.

Healthcare providers should help advocate for and implement national policies that support early identification and registration of adolescent pregnancies, ensure the availability of youth-sensitive antenatal and childbirth services, and enhance capacity building for healthcare providers.

Further research should expand on this observational study by examining contributing factors to adverse obstetric and perinatal outcomes, such as access to antenatal care, smoking, nutritional status, and other factors. Additionally, research on late postpartum maternal and neonatal outcomes among adolescent mothers is necessary.

## Data Availability

The raw data supporting the conclusions of this article will be made available by the authors, without undue reservation.
